# One‐Step Synthesis of a Durable and Liquid‐Repellent Poly(dimethylsiloxane) Coating

**DOI:** 10.1002/adma.202100237

**Published:** 2021-05-06

**Authors:** Jie Liu, Yuling Sun, Xiaoteng Zhou, Xiaomei Li, Michael Kappl, Werner Steffen, Hans‐Jürgen Butt

**Affiliations:** ^1^ Max Planck Institute for Polymer Research Ackermannweg 10 Mainz D‐55128 Germany

**Keywords:** adhesion, liquid‐repellency, poly(dimethylsiloxane), polymer brushes, wetting

## Abstract

Coatings with low sliding angles for liquid drops have a broad range of applications. However, it remains a challenge to have a fast, easy, and universal preparation method for coatings that are long‐term stable, robust, and environmentally friendly. Here, a one‐step grafting‐from approach is reported for poly(dimethylsiloxane) (PDMS) brushes on surfaces through spontaneous polymerization of dichlorodimethylsilane fulfilling all these requirements. Drops of a variety of liquids slide off at tilt angles below 5°. This non‐stick coating with autophobicity can reduce the waste of water and solvents in cleaning. The strong covalent attachment of the PDMS brush to the substrate makes them mechanically robust and UV‐tolerant. Their resistance to high temperatures and to droplet sliding erosion, combined with the low film thickness (≈8 nm) makes them ideal candidates to solve the long‐term degradation issues of coatings for heat‐transfer surfaces.

## Graphical abstract

A non‐stick coating with autophobicity is fabricated by a one‐step grafting‐from approach for poly(dimethylsiloxane) (PDMS) brushes on surfaces. Independent of surface tension, liquid droplets can easily slide on such surfaces.

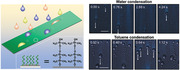

Rapid removal of liquid droplets from surfaces is of great concern in practical applications and fundamental research such as water harvesting,^[^ ^1^ ^]^ heat transfer,^[^ ^2^ ^]^ liquid manipulation,^[^ ^3^ ^]^ and so on. For drops to slide off surfaces easily even at low tilt angles, a low contact angle hysteresis is required. Very low roll‐off angles can be found on super‐liquid‐repellent surfaces. Combination of topographic features and low surface energy chemistry, in particular combining re‐entrant or double re‐entrant textures with surface fluorination, has been the typical strategy for fabricating super‐liquid‐repellent surfaces.^[^ ^4^ ^]^ This strategy requires that liquids are supported on topographic features and an air layer is maintained underneath. This so‐called Cassie state is, however, only metastable and can collapse under external forces leading to a Cassie‐to‐Wenzel transition. Furthermore, the surface textures tend to be fragile and the frequent use of fluorinated chemicals to decrease the surface energy raises concerns on their health and environmental impact.^[^ ^5^ ^]^ Another type of liquid‐repellent surface, slippery liquid‐infused porous surface (SLIPS), repels liquids through dynamic liquid/liquid/vapor contact‐line motion.^[^ ^6^ ^]^ The required slippery liquid must be both immiscible with and not be leeched out by the contacting liquid medium to avoid lubricant loss and contamination. Ensuring the long‐term robustness of such coatings and their wetting performance remains challenging.^[^ ^7^ ^]^ Therefore, other methods to create surfaces with good liquid repellency are desirable.

An alternative strategy, covalently attaching flexible macromolecules brushes such as PDMS and perfluorinated polyether onto smooth surface was proposed to repel liquids.^[^ ^8^ ^]^ The idea is that the high mobility of the flexible macromolecules allows them to act as a liquid‐like lubricating layer to liquids with a broad range of surface tensions.^[^ ^8c^ ^]^ Due to the covalent attachment to the surface, these molecular structures cannot be dissolved or displaced by the contacting liquids. Specifically, surfaces coated with PDMS brushes exhibit excellent resistance to high temperature treatment, photodegradation, and even scratching.^[^ ^8^, ^9^ ^]^ In addition, since the layers are only a few nanometers thick, they are transparent, do not influence the appearance of coated surfaces,and have little impact on heat conductivity. Preparation of PDMS brushes can be traced back to 1970, when Vermeulen et al. deposited a low‐liquid‐adhesion PDMS brush layer on glass surface with a vapor‐phase reaction for 16 h.^[^ ^10^ ^]^ However, grafting polymers from surfaces is generally based on complex and time‐consuming preparative procedures, limiting their use in practical applications.

McCarthy et al. systematically investigated new strategies to fabricate PDMS brushes on surfaces.^[^ ^11^ ^]^ They proposed to use dimethyldimethoxysilane (DMDMS) as monomer to polymerize PDMS brushes with sulfuric acid as catalyst.^[^ ^8a^ ^]^ After rinsing the surface with a copious amount of solvent to remove residual oligomer and acid, PDMS brushes with low liquid adhesion form on the silicon (or glass) surface after drying the reactive solution (includes DMDMS, sulfuric acid, and isopropyl alcohol) for some time. Compared to McCarthy's method, we developed a simpler approach to graft PDMS brushes on surfaces with no catalyst required. Furthermore, we characterized the stability of PDMS brushes under tape‐peeling, sonication, drop sliding corrosion, heating, UV degradation, acid corrosion, and more. McCarthy et al. only investigated the effect of heating at 100 °C.

Here, we report a further simplified processing method, using a fast one‐step approach to prepare PDMS brushes that exhibit autophobicity and low contact angle hysteresis for a broad range of liquids. PDMS brushes are synthesized directly on the desired surface through polymerization of reactive silane monomers (dichlorodimethylsilane, DCDMS) without initiator. Polymerization of DCDMS happens under hydrolysis of water.^[^ ^12^ ^]^ Reacting with water, the Si—Cl group of DCDMS is hydrolyzed to Si—OH. When a silicon wafer pretreated with oxygen plasma is rinsed in DCDMS/toluene solution, DCDMS attaches spontaneously and fast to the surface via reaction of the Si—OH or Si—Cl group with hydroxyl groups on the surface. Further condensation reactions between Si—OH and Si—OH or Si—Cl at the surface will follow, finally forming PDMS brushes (**Figure** **1**a).^[^ ^13^ ^]^ The high reactivity of DCDMS promotes the fast grafting process of PDMS brush.

**Figure 1:**
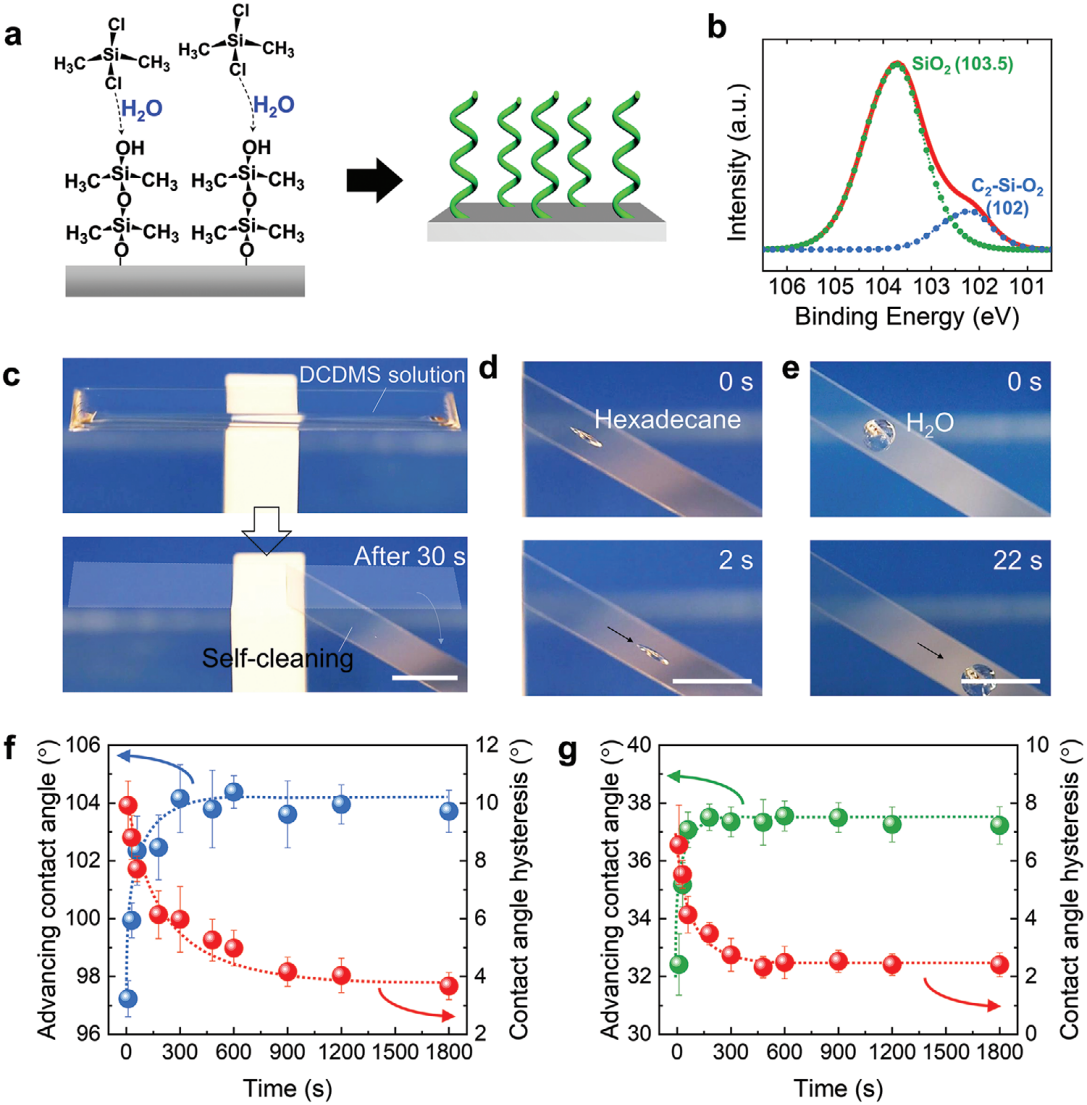
Ultrafast processing of PDMS brushes. a) Schematic illustration of the formation of PDMS brushes from dichlorodimethylsilane (DCDMS) monomers. b) Si 2p peaks (red line) measured by X‐ray photoelectron spectroscopy (XPS) of the PDMS brushes grafted onto a silicon wafer. The fit indicates the presence of O—Si—O (green line) and —O—Si(CH_3_)_2_—O— (blue line) bonds. Reaction time: 30 min. The sample was washed with toluene for five times. c) Autophobicity of PDMS brushes on glass: after 30 s grafting time, the reactant can be fully removed by simple tilting. d) Sliding of *n*‐hexadecane on the PDMS‐brushes‐coated glass slide. Liquid volume: 5 µL. e) Sliding of water on the PDMS‐brushes‐coated glass slide. Liquid volume: 10 µL. Scale bar (c–e): 1 cm. f) Advancing contact angle (*Θ*_ACA_) and contact angle hysteresis (∆*Θ*) of water as function of grafting time. g) Advancing contact angle and contact angle hysteresis of *n*‐hexadecane as function of grafting time.

With this recipe, PDMS brushes were fabricated by immersing the substrate, in our case silicon wafer (used as substrate in the following if not otherwise stated) or glass, into a toluene solution containing DCDMS (0.24 × 10^−3^ m). We maintained a saturated concentration (*c*_water_ ≈ 0.024 × 10^−3^ m) of water in toluene at room temperature. X‐ray photoelectron spectroscopy (XPS) of PDMS brushes on silicon wafers revealed the existence of the —O—Si(CH_3_)_2_—O— (≈102 eV) in addition to the previously existing SiO_2_ (Figure 1b). This proves the successful grafting of poly(dimethylsiloxane) (PDMS) molecules. Grafting‐from of brushes occurs synchronously on the whole surfaces in a homogeneous solution, therefore the final surface is smooth with low roughness; AFM images of PDMS surface show a roughness of Ra ≈ 0.1 nm (Figure S1, Supporting Information).

Conveniently, the PDMS brushes are autophobic in the sense that the reactant solution itself can be easily removed. When for example coating a glass slide by covering its top with DCDMS solution for 30 s (Figure 1c), the reactant solution was easily removed by simply tilting the surface. No residual stains were left (Movie S1 and Figure S2, Supporting Information). The induced self‐repellency toward the reactant solution implies that no extra washing step is necessary to clean the surface, which reduces solvent waste. The fast coating process makes the glass surface repellent to liquids with both low (hexadecane) and high (water) surface tensions. “Repellent” here means that hexadecane and water droplets easily run off a tilted surface (Figure 1d,e).

The advancing (*Θ*_ACA_) and receding (*Θ*_RCA_) contact angles as well as contact angle hysteresis (∆*Θ* = *Θ*_ACA_ – *Θ*_RCA_) of water and hexadecane drops were used to illustrate the liquid repellency (Figure 1f,g). Within a reaction time of 30 s, the contact angle hysteresis of the silicon wafer decreased to ∆*Θ* = 9° for water and *∆Θ* = 5° for hexadecane (water: *Θ*_ACA_ = 100°, *Θ*_RCA_ = 91° and hexadecane: *Θ*_ACA_ = 35°, *Θ*_RCA_ = 30°). For a longer grafting time (>15 min), PDMS brushes become thicker (Figure S3, Supporting Information) and the contact angle hysteresis of water was reduced to less than 5°. According to the stretching length (*L*) of the PDMS molecules, the molecular weight of the brushes can be roughly estimated. The length of —Si—O— is around 1.61 Å. By dividing the stretching length by the length per monomer we get the minimal number of monomers per chain. When the grafting time is 30 min (*L* = 13 ± 1 nm), the molecular weight of the PDMS brushes must be larger than 3000 g mol^−1^. The reaction rate can be accelerated by increasing the concentration of DCDMS (Figure S4, Supporting Information). It turned out that the water concentration plays an important role in the grafting process. The higher concentration of water can effectively accelerate the reaction and reduce grafting time (Figure S5, Supporting Information).

Contact angle hysteresis has an important physical significance: It is directly linked to the lateral adhesion and the tilt angle α required to let the drop slide off the surface:^[^ ^14^ ^]^

(1) $$\sin\alpha \;\;=\;\;\frac{kw\gamma }{gV\rho }\left(\cos\Theta_{\text{RCA}}-\cos\Theta_{\text{ACA}}\right)$$

Here, *k* ≈ 1 is a geometrical factor, *w* is the width of the contact area of the drop, γ is the surface tension of the liquid, *g* = 9.81 m s^−2^ is the acceleration of gravity and ρ is the density of the liquid. Since contact angle hysteresis is relatively low, drop shapes of small drops can be well described by a spherical cap model. In this aspect, a natural length scale for drops is the capillary length $\kappa \;\;=\;\;\sqrt{\gamma \text{/g}\rho }$, which for water is 2.7 mm. In the spherical drop model, the width of the drop is twice the contact radius *a*. The contact radius is given by the drop volume: $\alpha \;\;=\;\;\sin\Theta {\left(\frac{3V}{\pi \beta }\right)}^{1/3}$ with β = (1 – cos*Θ*)^2^ (2 + cos*Θ*). Here *Θ* is the mean contact angle. Therefore,

(2) $$\sin\alpha \;\;=\;\;\frac{2\gamma \sin\Theta }{gV^{2/3}\rho }{\left(\frac{3}{\pi \beta }\right)}^{1/3}\left(\cos\Theta_{\text{RCA}}-\cos\Theta_{\text{ACA}}\right)$$

That implies that reducing the contact angle hysteresis decreases the lateral adhesion and thus the sliding angle. Due to the *V* ^−2/3^ scaling, large drops easily slide down tilted planes while small drops tend to stick. Therefore, we used small drops (5 µL) to test our surfaces.

Silicon wafers coated with PDMS brushes show low sliding angles for liquids with a broad range of surface tensions. An *n*‐hexane (γ = 18.4 mN m^−1^) sessile drop slides down from the PDMS brushes tilted by 2° (**Figure** **2**a). An ethanol (γ = 22.1 mN m^−1^) and a toluene (γ = 28.4 mN m^−1^) droplet slide easily off the surface with a tilting angle of 5°. For comparison, a surface coated with 1H,1H,2H,2H‐perfluorooctyltrimethoxysilane shows a higher lateral adhesion to liquids than PDMS brushes: an *n*‐hexadecane droplet slides on PDMS brushes but remains stationary on a fluorinated surface when α = 5° (Figure S6, Supporting Information). The lateral adhesion forces (*f*) of water droplets (5 µL) on PDMS brushes (*f* = 18.9 ± 1 µN) was much lower than that on the fluorinated surface (*f* = 60.0 ± 3 µN) when the relative speed between droplet and surface was 250 µm/s (Figure S7, Supporting Information). Contact angle hysteresis measurements show that liquids such as ethanol, isopropyl alcohol (23.0 mN m^−1^), hexadecane (27.5 mN m^−1^), toluene, dimethylformamide (DMF) (37.1 mN m^−1^), dimethyl sulfoxide (DMSO) (43.5 mN m^−1^), diiodomethane (50.8 mN m^−1^), and water (72.8 mN m^−1^) all present a low lateral adhesion to the surface with ∆*Θ* < 5° (Figure 2b). The sliding angles of the liquid droplets are studied versus the volume (5, 10, and 20 µL) (Figure 2c). 10 µL droplets independent of the type of liquid slide at α < 5°. The roll‐off angles observed for liquids with different surface tension and drops with different volumes agree with theoretical predictions of Equation (2). The PDMS‐brush‐coated surface repels low and high viscosity liquids such as poly(propylene glycol) (Mn ≈ 725, viscosity: 115 cP), poly(ethylene glycol)‐*block*‐poly(propylene glycol)‐*block*‐poly(ethylene glycol) (*M*_n_ ≈ 2000, viscosity: 325 cP), and poly(ethylene glycol)‐*block*‐poly(propylene glycol)‐*block*‐poly(ethylene glycol) (*M*_n_ ≈ 4400, viscosity: 1200 cP) (Figure S8, Supporting Information).

**Figure 2:**
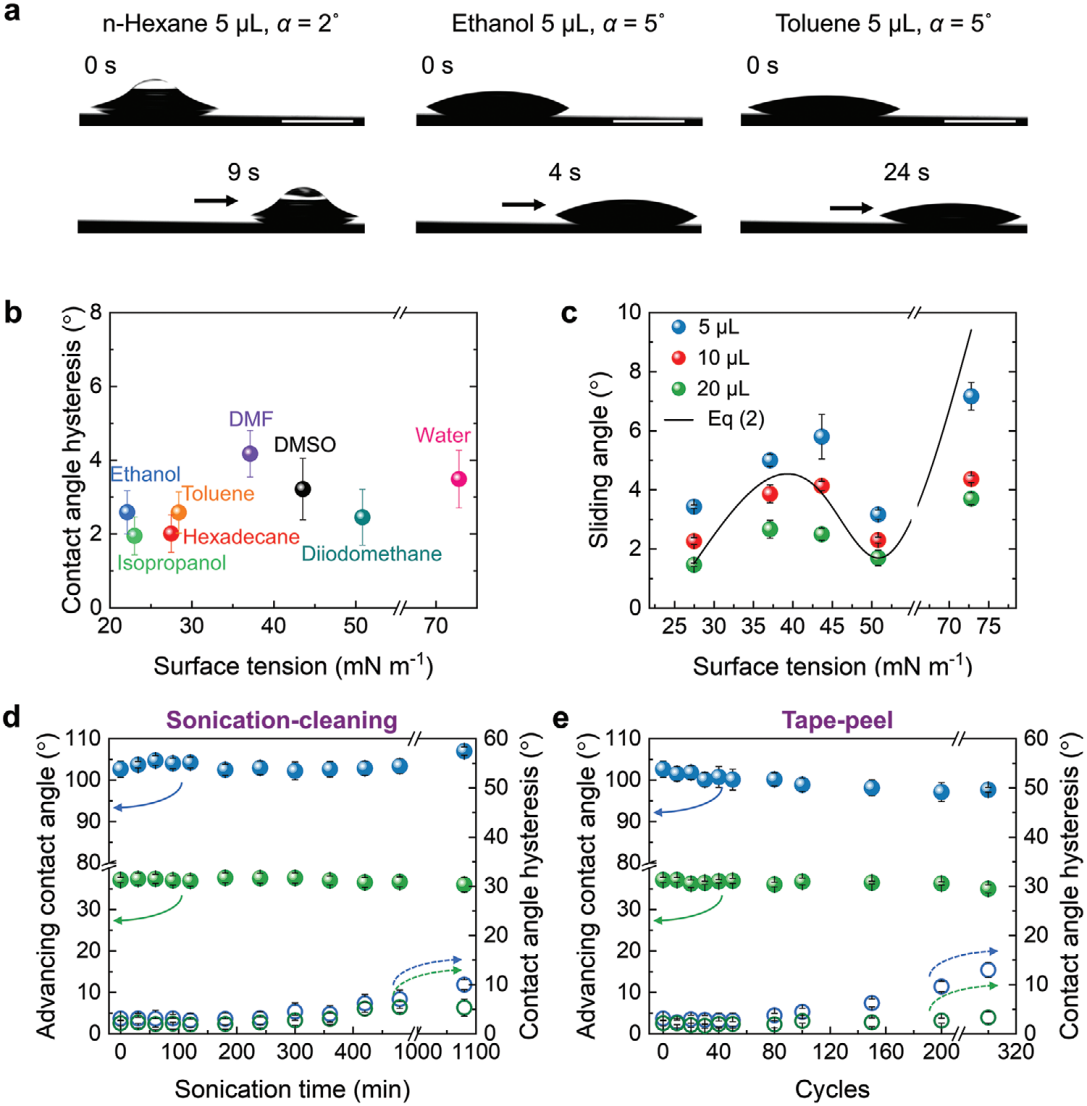
Robust liquid‐repellent PDMS brushes. a) Time‐sequence images of hexane (left), ethanol (middle), and toluene (right) droplets sliding down a tilted silicone brush surface. To increase visibility of the hexane drop, a small air bubble was injected on the top of hexane droplet. Scale bar: 2 mm. b) Contact angle hysteresis of various liquids on PDMS brushes. Grafting time: 30 min. c) Sliding angle of drops of various liquids (from left to right: *n*‐hexadecane, DMF, DMSO, diiodomethane, water) on PDMS brushes. The black line shows sliding angles of liquid drops (10 µL) versus surface tension plotted according to Equation (2). The mean, advancing, and receding contact angles were measured values of the five kinds of liquids. d) Water (*Θ*_ACA_: 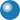 and ∆*Θ*: 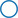) and *n*‐hexadecane (*Θ*_ACA_: 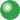 and ∆*Θ*: 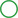) repellency of the PDMS brushes versus sonication‐washing time. e) Water (*Θ*_ACA_: 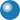 and ∆*Θ*: 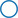) and *n*‐hexadecane (*Θ*_ACA_: 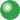 and ∆*Θ*: 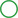) repellency of the PDMS brushes resisting tape‐peeling tests.

For practical applications, robustness and durability of surface coatings is of critical importance. In a first test, sonication (45 kHz, 60 W) of our grafted PDMS brushes for 6 h in toluene did not alter the wetting properties for both water and hexadecane (Figure 2d). This resistance to sonication cleaning excludes the possibility that the low adhesion is caused by embedded, non‐grafted silicone monomer or oligomer.^[^ ^9^, ^15^ ^]^ The contact angle hysteresis of water and hexadecane on the PDMS brushes starts to slightly increase after 7 h of sonication. Nevertheless, the hysteresis is still not larger than 10° even after 18 h sonication. In contrast, on a 1H,1H,2H,2H‐perfluorodecyltriethoxysilane modified surface contact angle hysteresis increased from 18° to 29° after 1 h sonication (Figure S9a, Supporting Information).

In a second test, a Scotch tape (3M 810) was homogeneously pressed onto a PDMS brush grafted silicon wafer (load: 11.5 kPa, duration: 20 s) and then peeled off (Figure 2e). The peeling force (*F*_peeling_) of the tape on silicon wafer (*F*_peeling_ = 110 ± 12 N m^−1^) was markedly reduced by PDMS brushes (*F*_peeling_ = 4 ± 1 N m^−1^). Eighty adhesion–peeling cycles did not influence the repellency of both water and hexadecane; the contact angle hysteresis remained below 5° for both liquids. After 300 peel‐test cycles, the advancing and receding contact angles of water decreased from 103° and 99° to 97° and 84°, respectively; contact angle hysteresis increased from 4° to 13°. In contrast, wetting performance for hexadecane was not altered even after 300 peel‐tests. Compared to the mechanical stability of the PDMS brushes, the fluorinated surface rapidly lost their hydrophobicity. The receding contact angle of water decreased from 99° to 78° and the contact angle hysteresis increased from 18° to 25° after 100 peel‐tests (Figure S9b, Supporting Information).

A temperature‐tolerance test was carried out to study the durability of our PDMS brushes (**Figure** **3**a,b) for heat transfer applications. The coating can resist long term heat treatment at 100 °C in air, as advancing and receding contact angles of water and hexadecane on PDMS brushes remained constant for at least 32 days. When the temperature was increased to 250 °C, the advancing contact angle of water increased from 104° to 107° and remained constant for 15 days. ∆*Θ* of water increased slightly during this time, reaching 8° after 15 days of heat treatment. So even at such high temperatures, little loss of wetting properties was observed. Contact angles changed more in the case of *n*‐hexadecane but contact angle hysteresis still remained very low (∆*Θ* = 3°). The decrease of the contact angle of *n*‐hexadecane might be caused by the reaction between end groups (—OH) at 250 °C, causing the loss of the molecular mobility of the brushes.^[^ ^8^, ^11^ ^]^

**Figure 3:**
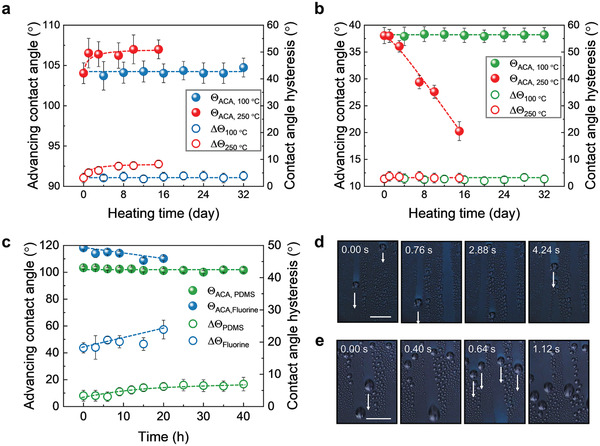
Durability of the PDMS brushes under high‐temperature and water vapor treatment. a) Water repellency of the PDMS brushes versus aging time at 100 °C and 250 °C. b) Hexadecane repellency of the PDMS brushes versus aging time at 100 °C and 250 °C. c) Durability of wetting property of water on PDMS brushes and fluorinated surface under water vapor treatment at 70 °C. d) Image sequence shows sliding of condensed water droplets during water vapor treatment. Vapor temperature: 70 °C. e) Image sequence shows sliding of condensed toluene droplets during toluene vapor treatment. Vapor temperature: 70 °C. Scale bar in (d,e): 0.5 cm.

In heat transfer, especially condensation heat‐transfer, maintenance of a low lateral adhesion of droplets is a key factor to enhance condensation rates.^[^ ^2a^ ^]^ The PDMS brushes exhibit a constant and low water adhesion independent of temperature in a wide temperature window (0 °C to 70 °C) (Figure S10, Supporting Information). A droplet sliding erosion test was designed to characterize the durability of PDMS brushes under high temperature (70 °C) water vapor treatment (Figure S11, Supporting Information). The water vapor was prepared by heating water at 200 °C to obtain water steam, and then the steam was cooled to 70 °C on the surfaces during which condensed droplets formed. As shown in Figure 3c, even after 40 h of treatment (flow rate: 0.8 ± 0.1 L min^−1^), the advancing contact angle of water on the PDMS brushes did not change. Only the contact angle hysteresis increased from 4° to 7°. For the 1H,1H,H,2H‐perfluorooctyltrimethoxysilane coated surface as a control, the initial contact angle hysteresis was much higher (∆*Θ* = 18°). After 20 h of treatment, the advancing contact angles decreased from 118° to 109°, accompanied with the contact angle hysteresis increase from 18° to 24°. This deterioration indicates degradation or partial removal of the fluorination layer under the sliding of condensing water droplets. The reason is that the fluorinated surface has a much higher adhesion to water droplets than the PDMS brushes. This leads to a higher peeling force loaded to the fluorinated molecules from water droplets; thus the molecules are more easily detached from the surface. In addition, the higher thickness of the PDMS brush layer ensures its better stability than the fluorinated layer. Thus, our PDMS brushes are significantly more stable than fluorinated silane coated surfaces.

Condensed water droplets are expected to move easily due to the low hysteresis of water on PDMS brushes. Rather than reporting the tilt angle at defined volume, we measured the sizes of water droplets on different vertical surfaces (α = 90° fixed tilt angle) such as PDMS brushes, silicone oil impregnated PDMS brush surface (liquid‐impregnated surface, SLIPS), and fluorinated surface (Figure S12a, Supporting Information). Condensed water droplets slid off the vertical PDMS brushes with a diameter (*D*) around 1.2 mm, a little bit larger than that on SLIPS (*D* = 1.0 mm). In contrast, the minimum sliding size of water drops on fluorinated surface is much bigger (*D* = 2.3 mm). Therefore, the mass transfer efficiency of water on these three surfaces should differ greatly. Water‐collection efficiency (water volume slid off per square meter surface) was measured when we cooled the surfaces to 0 °C (room temperature: 20 ± 1 °C, relative humidity: 80 ± 5%) (Figure S12b, Supporting Information). PDMS brushes present the similar water‐collection efficiency with the SLIPS in 1 h. Cloaking of the droplets by the oil on SLIPS reduces the coalescence of water drops^[^ ^16^ ^]^, resulting in condensed droplets sliding earlier on PDMS brushes than on SLIPS. Requiring a comparatively large drop size for sliding (Figure S12a, Supporting Information), no water was collected on fluorinated surfaces within 1 h condensation time as the droplet remained pinned.

Depending on application, not only water may be used as heat transfer medium. Due to their excellent liquid repellency to a broad range of liquids, PDMS‐brush‐coated surfaces could be used in heat and mass transfer in different situations.^[^ ^17^ ^]^ Besides repellency to condensate water (Figure 3d), condensed liquids with low‐surface‐tension such as toluene can also slide fast and easily on the PDMS brushes (Figure 3e, Movie S2, Supporting Information).

The fast and spontaneous grafting reaction of PDMS brushes on surfaces allows using various coating methods such as dip‐coating, drop‐casting, or spray coating to form PDMS brushes. We applied the reaction solution with a soaked textile or paper to modify large areas in a controlled way (**Figure** **4**a). As an example, a paper (80 g m^−2^) was used to hold the solution and spread the coating solution on the surface. In this way, the coating area was increased 16 times compared to the drop‐casting method. After grafting PDMS brush for 3 min, toluene droplets rapidly slid on the surface (Figure S13, Supporting Information).

**Figure 4:**
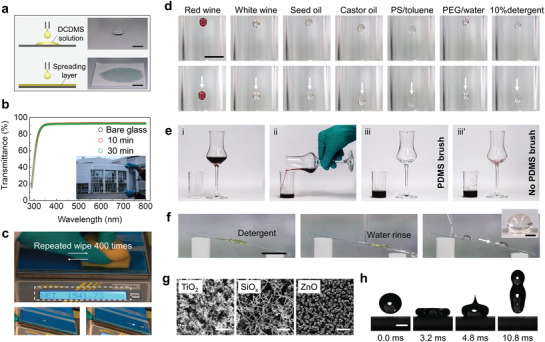
Applications of PDMS brushes. a) A window glass plate (soda‐lime glass, 20 × 10 cm^2^) was covered with a piece of commercial paper (80 g m^−2^). Then the reactant solution (200 µL) was applied. The paper was used to hold the reaction solution and promote its spreading on the surface. Scale bar: 2 cm. b) UV–vis spectra show the film transmittance as a function of modification time. The inset shows a window glass (30 × 20 cm^2^) coated for 30 min. c) Wearing tolerance of PDMS brushes on window glass (15 × 7 cm^2^). Grafting time: 3 min. d) Images show sliding of various liquids on coated glass. Liquids from left to right: red wine, white wine, seed oil, castor oil, polystyrene solution in toluene (1 wt%), poly(ethylene glycol) aqueous solution (50 wt %), and detergent aqueous solution (10 wt%). Scale bar: 1 cm. e) Pouring of red wine from wine glass and no staining on the coated surface (iii). A layer of wine stayed on the glass surface after pouring (iii'). f) Washing of concentrated detergent (0.3 mL) on a coated glass with water flux (25 mL). Grafting time: 3 min. Scale bar: 2 cm. Inset shows a water drop after the detergent was removed. Scale bar: 2 mm. g) Metal‐oxide surfaces with nanostructures. Surfaces from left to right: titanium dioxide (TiO_2_) nanotexture, silicone nanofilament (SiO*_x_*), and zinc oxide nanopillars (ZnO). Scale bars (left to right): 500 nm, 500 nm, 1 µm. h) Impacting and bouncing of a 5 µL water droplet shows superhydrophobic performance of TiO_2_ hierarchical surface after grafting with PDMS brushes in 3 min. Dropping height: 2 cm. Scale bar: 1 mm.

Transparency of coatings is required in many applications.^[^ ^18^ ^]^ Good transparency of the PDMS brushes opens the possibility to coat surfaces without changing their outward appearance. The overlapped UV/Vis spectra of the glass (thickness: 1 mm) coated with PDMS brushes with different thickness indicates no transparency loss was caused by the brush layer (Figure 4b). This is mainly attributed to the nanoscale thickness of the brush layer and its refractive index close to glass (silicone oil: 1.4, window glass: 1.5).

Wear tolerance is a key feature in practical applications. One potential application we investigated preliminarily is preparing low‐adhesion and wash‐free liquid containers based on to the liquid‐repellent performance of PDMS brushes. To test how robust the surface is against wear, a piece of window glass (soda‐lime glass, size: 15 × 7 cm^2^) was coated with PDMS brushes with a reaction time of 3 min (Figure 4c). The coated window glass was subjected to wear by a hand‐held commercial scouring pad on a balance so that we can record the applied pressure during forward/backward stroke cycles. The average pressure during wearing test was 6.6 ± 1.2 kPa. After wearing the brush‐coated glass for 400 stroke cycles, water droplets can still easily slide on the surface. The PDMS‐brush‐coated surface kept its repellency to both water and *n*‐hexadecane even after it was slightly destroyed by rubbing with sandpaper (1000 mesh) under the pressure of 1.1 kPa (Figure S14, Supporting Information). In addition, a water jet with a velocity of 4.7 m s^−1^ was applied to impact the surface three times for 1 s (Figure S15, Supporting Information). After the treatment, the wetting property of the surface remains unchanged with ∆*Θ*_water_ = 4° ± 1° and ∆*Θ*_hexadecane_ = 2.7° ± 1°.

Liquid adhesion on surfaces causes waste of water or solvents for cleaning. A glass bottle coated with PDMS brushes presents low sliding adhesion to liquids such as wine, plant oil, polymer solutions, and detergent (Figure 4d). After coating a layer of PDMS brushes, red wine drops can easily slide down the surface (Figure 4e). This indicates that much less wine drops will stain the inner surface of the coated glass after drinking, and therefore reduces the waste of water in cleaning. In contrast, a thin layer of wine always stains the bare glass. The easy sliding of oils such as castor oil and seed oil reduces waste of cooking oil attached to their bottles (Figure 4d). In addition, cleaning of chemical reaction containers in the lab is a daily nuisance. Chemicals left on the surface need a lot of solvent to clean, because even little residues may spoil following reactions. This problem can be reduced by coating surface with PDMS. For example, glass coated with PDMS brushes presents good repellency to a polystyrene in toluene solution (1 wt%) and poly(ethylene glycol) (PEG) aqueous solution (50 wt%) (Figure 4d). The surface kept this antifouling benefit even when it was placed outdoor for 21 days (Figure S16, Supporting Information).

Furthermore, to clean dirty surfaces, a detergent solution is usually used. However, remaining stains of detergent after drying raise concerns on their health impact. Removing detergent stains requires a lot of water. We demonstrate that aqueous detergent solutions (10 wt%) easily slide off PDMS brushes (Figure 4d). Even when a coated glass was stained with a concentrated detergent, it can be completely cleaned by washing with a water flow (Figure 4f; and Movie S3, Supporting Information). The complete removal of the detergent from the surface was inferred from the shape (*Θ* ≈ 101°) of water drop and fast sliding of water drops on the surface after washing. It is noted that the mass of water needed to clean detergent on PDMS brushes is maximum 50% of that needed to clean hydrophilic glass surface, and it is hard to know if the hydrophilic surface is clean completely or not. Surfaces coated with PDMS brushes also present tolerance to UV illumination (Figure S17, Supporting Information), making them especially suitable for outdoor applications. Its resistance to acid benefits its application in corrosion resistance (Figure S18, Supporting Information).

PDMS brushes can become a substitute to decrease surface energy also of other materials than glass. Three metal oxide surfaces such as titanium dioxide (TiO_2_) nanotexture, silicon oxide (SiO*_x_*) nanofilament, and zinc oxide (ZnO) nanorod were prepared according to previous studies.^[^ ^9^, ^19^ ^]^ All these surfaces are easily and rapidly coated with PDMS brushes and became superhydrophobic in 3 min (Figure 4g). After modification, water drops easily rebound completely from such surfaces (Figure 4h). However, this method still has shortcomings. Since surface hydroxyl groups are required, and since HCl is produced during synthesis, it is of limited use on wood and metals such as steel, copper or gold (Figure S19, Supporting Information). Only aluminum, with its oxide layer, becomes moderately hydrophobic.

In conclusion, a fast one‐step approach to coat poly(dimethylsiloxane) brushes through direct polymerization of dichlorodimethylsilane on surfaces was reported. This cheap and green coating exhibits low lateral adhesion to liquids including the reaction solution itself. To reduce the amount of material required to form a PDMS brush, we hold the reaction solution by a porous spreading layer (a paper). Using paper also allows coating of defined areas. The autophobic performance provides possibilities to apply PDMS brush coatings to liquid containers, which avoids loss of products due to incomplete emptying and reduces waste of water and solvents for subsequent cleaning. The strong tolerance of PDMS brushes to high temperature treatment and UV illumination as well as wear resistance makes them suitable to prolong the service time of hydrophobic surfaces used in heat transfer.

## Experimental Section

### Fabrication and Characterization of the PDMS Brushes

Dichlorodimethylsilane (DCDMS, Sigma–Aldrich) was dissolved in 40 mL of toluene. The toluene was saturated with water (0.024 × 10^−3^ m). The molar ratios of DCDMS to water were controlled to be 5:1, 10:1, and 20:1 to determine the best reaction condition. The solution was swirled to mix for about 30 s and allowed to stand for 5 min at room temperature before use. Oxygen‐plasma‐treated silicon wafer (10 cm diameter, N‐type doped with phosphor, (100) oriented, 525 µm thick) or soda‐lime glass was submerged in the reactive solution for a certain time (from 30 s to 1 h) to get PDMS‐brush‐coated surfaces. The wetting properties of the surfaces were investigated using a contact angle measurement device equipped with a side camera (IDS uEye camera) and a goniometer. XPS was conducted using a Kratos Axis UltraDLD spectrometer (Kratos, Manchester, UK) to characterize the element composition of the silicone brush.

### Fabrication of the Fluorinated Surfaces and Liquid‐Impregnated Surfaces

An oxygen‐plasma‐treated silicon wafer was coated with 1H,1H,2H,H‐perfluorooctyltrimethoxysilane (Sigma–Aldrich) under vacuum for 12 h. The surface was further heat treated at 120 °C for 2 h to obtain hydrophobicity. Liquid‐impregnated surfaces were prepared by spin‐coating (4000 rpm, time: 60 s) a layer of silicone oil (100 cSt, 10 wt% in hexane, Sigma–Aldrich) on PDMS brushes (grafting for 30 min).

### Droplet‐Sliding Erosion Assays

A dual control system was established to characterize the durability of PDMS brushes under high temperature (70 °C) and high humidity water vapor treatment (Figure S11, Supporting Information). A nitrogen flow was passed through boiling water in a bottle with one inlet and one outlet. The wet nitrogen was heated to 200 °C in a spiral circular copper tube. Surfaces (fluorinated surface and PDMS brushes) were treated with the heated water vapor. By changing the distance between surface and outlet, the vapor temperature can be controlled. The temperature of the vapor was monitored by a mercury thermometer with measuring range of 200 °C. The durability of the surfaces was demonstrated by measuring the wetting performance of the surfaces with different treating time.

### Coating PDMS Brushes on Various Surfaces

Titanium dioxide (TiO_2_) hierarchical surfaces (composed of 99 wt% TiO_2_ and 1 wt% SiO_2_) were prepared via liquid flame spray.^[^ ^19a^ ^]^ Hydrogen (50 L min^−1^) and oxygen (15 L min^−1^) were used as combustion gases to achieve a turbulent, high temperature flame (>2500 °C). Tetraethyl orthosilicate (TEOS, 98% pure, Alfa Aesar) and titanium (IV) isopropoxide (TTIP, 97% pure, Alfa Aesar) were dissolved in isopropyl alcohol. The overall Si + Ti atomic concentration in the precursor solution was 50 mg mL^−1^. The ratio of silicon to titanium was 1/99 in precursor solution. Silicone nanofilament was prepared from trichloromethylsilane (TCMS).^[^ ^19b^ ^]^ After immersing an oxygen‐plasma‐treated glass in TCMS solution in toluene (water content: 260 ppm) for 6 h, the surface was coated with nanofilaments. The surface was further washed with hexane three times. The ZnO nanorod surfaces were prepared by spin coating (speed: 4000 rpm, time: 60 s) of zinc acetate dihydrate (0.75 m) in 2‐methoxyethanol and monoethanolamine (0.75 m).^[^ ^9b^ ^]^ Sintered the surface at 350 °C for 30 min in order to get a ZnO thin layer. ZnO nanorods were further grown for 2 h in an aqueous solution containing zinc nitrate (0.025 m) and hexamethylenetetramine (0.025 m) at 90 °C. PDMS brushes were coated for 3 min after oxygen plasma treatment. After oxygen plasma treatment, all the surfaces were coated with PDMS brushes.

## Conflict of Interest

The authors declare no conflict of interest.

## Author Contributions

J.L. and Y.S. contributed equally to this work. J.L., Y.S., and H.‐J.B. designed and performed research; J.L., Y.S., X.Z., X.L., and M.K. contributed new reagents/analytic tools; J.L. and Y.S. analyzed data; J.L., M.K., W.S. and H.‐J.B. wrote the paper. All authors have given approval to the final version of the manuscript.

## Supporting information

Supporting Information

Supplemental Movie 1

Supplemental Movie 2

Supplemental Movie 3

## Data Availability

The data that support the findings of this study are available from the corresponding author upon reasonable request.
