# Supplementary material for: One‐Step Synthesis of a Durable and Liquid‐Repellent Poly(dimethylsiloxane) Coating
Source: Adv Mater. 2021 May 6;33(23):2100237. doi: 10.1002/adma.202100237 (PMC11468872; doi:10.1002/adma.202100237)
Supplement: Supplementary file 1 — Supporting Information [file ADMA-33-2100237-s001.pdf]

© 2021 Wiley-VCH GmbH

ADVANCED MATERIALS

Supporting Information

for *Adv. Mater.,* DOI: 10.1002/adma.202100237

One-Step Synthesis of a Durable and Liquid-Repellent Poly(dimethylsiloxane) Coating

Jie Liu, Yuling Sun, Xiaoteng Zhou, Xiaomei Li, Michael Kappl, Werner Steffen, and Hans-Jürgen Butt**

One-Step Synthesis of a Durable and Liquid-Repellent Poly(dimethylsiloxane) Coating

Jie Liu[1,*], Yuling Sun[1], Xiaoteng Zhou, Xiaomei Li, Michael Kappl, Werner Steffen, Hans-Jürgen Butt*

Max Planck Institute for Polymer Research, Ackermannweg 10, D-55128, Mainz, Germany.

[1]These authors contributed equally: Jie Liu and Yuling Sun.

*E-mail: liujie@mpip-mainz.mpg.de; butt@mpip-mainz.mpg.de

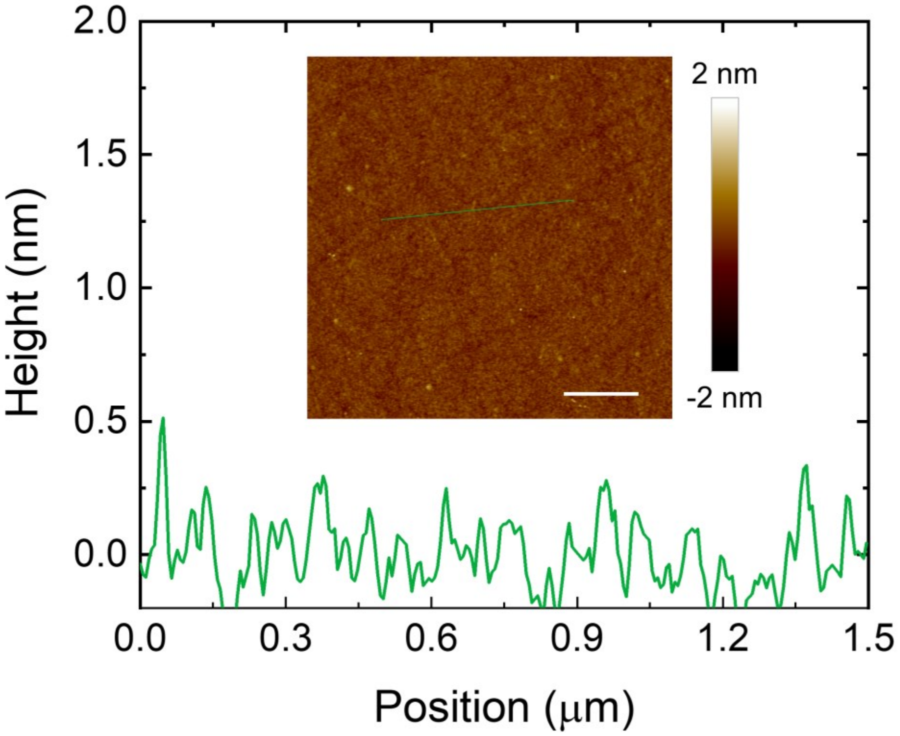

Figure S1 Morphology of PDMS brushes on silicon wafer. Image measured by atomic force microscope (AFM) in tapping mode. This shows the smooth surface of PDMS brushes with a low roughness of 0.1 nm (Ra) over an area of 3 × 3 μm^2. Scale bar: 600 nm. Reaction time: 30 min. The vertical profile of the PDMS along the labelled green line indicates a homogeneous PDMS brush layer formed on silicon wafer.

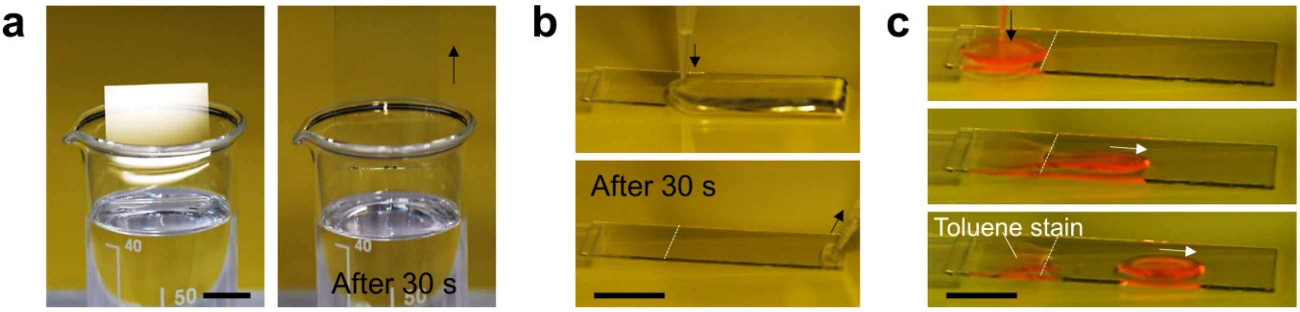

Figure S2 Easy fabrication of PDMS brushes. a) Fabrication of PDMS brushes on a glass slide by dip-coating. No DCDMS solution stains stay on the glass slide when pulling it from the solution after coating for 30 s. Scale bar: 1 cm. b) Fabrication of PDMS brushes on a glass slide by drop-casting. The reactive solution was easily removed without leaving stains. Scale bar: 2 cm. c) Images show toluene sliding on PDMS brush coated glass. In contrast to coated part, placement of drop occurs on upper, uncoated part. Toluene was labelled with Nile red. Scale bar: 2 cm.

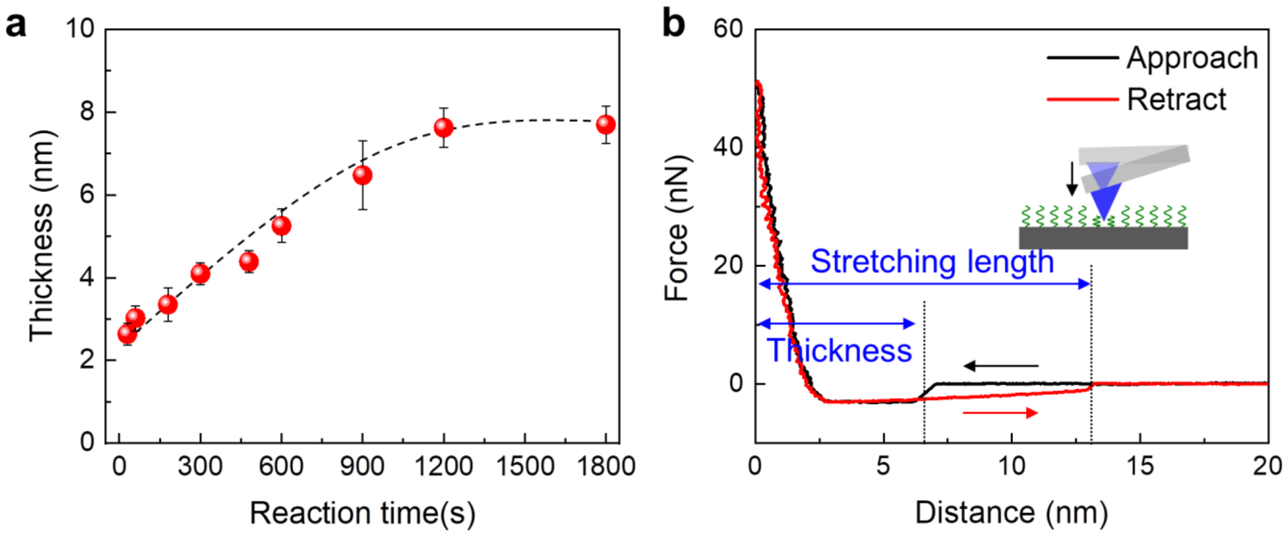

Figure S3 Thickness evolution of the PDMS brushes versus grafting time. a) The thickness of PDMS brushes on silicon wafers can be controlled from 2 nm to 8 nm. Each data point was calculated from 200 measurements. b) The thickness of PDMS brushes was extracted from force spectroscopy measurements with an atomic force microscope (NanoWizard IV, JPK Instruments, Berlin, Germany)[1]. The cantilever mounted on the piezoelectric translator of the AFM can be controlled to approach or retract from surface with a constant velocity. The deflection of the cantilever was measured as a function of the piezo displacement with a split photodiode during approach and retraction. The resulting detector signal versus piezo position curves were converted to force versus distance curves (force curves) by the following procedure: first, a linear fit of the zero-force line was subtracted from the force curve. Second, the cantilever deflection sensitivity was determined by fitting the linear compliance region of the force curve corresponding to the portion of the measurement during probe contacting with a rigid substrate (clean silicon wafer without PDMS brush). We subsequently obtained force curves by subtracting the cantilever deflection from the piezo position to obtain correct position values and multiplying the deflection (obtained by dividing the detector signal value by the cantilever deflection sensitivity) by the spring constant of the cantilever to obtain force values. The black line shows the force between the probe and surface while approaching. At a distance of about 6-7 nm, an attractive force is observed, leading to negative

deflection of the cantilever. This event indicates the contact between tip and upper end of the PDMS layer, leading to attractive van der Waals and capillary forces between tip and layer. As the tip pushes further into the layer, forces become more and more repulsive, until a hard contact is reached (vertical force line). The PDMS brush thickness was determined as the distance between jump-in event and hard contact. At the same time, when the tip presses the PDMS brushes, some PDMS molecules can physically be attached to the tip. Recording the force loaded on the tip when withdrawing the tip from the surface, a stretching length (L) of PDMS molecules was defined, $L = 13 \pm 1$ nm. We assume that the molecular chain is completely straightened under stretching. The length of –Si–O– is around 1.61 Å. By dividing the stretching length by the length per monomer we get the minimal number of monomers per chain. Therefore, the molecular weight of the PDMS brushes must be larger than 3000 ± 200 g/mol.

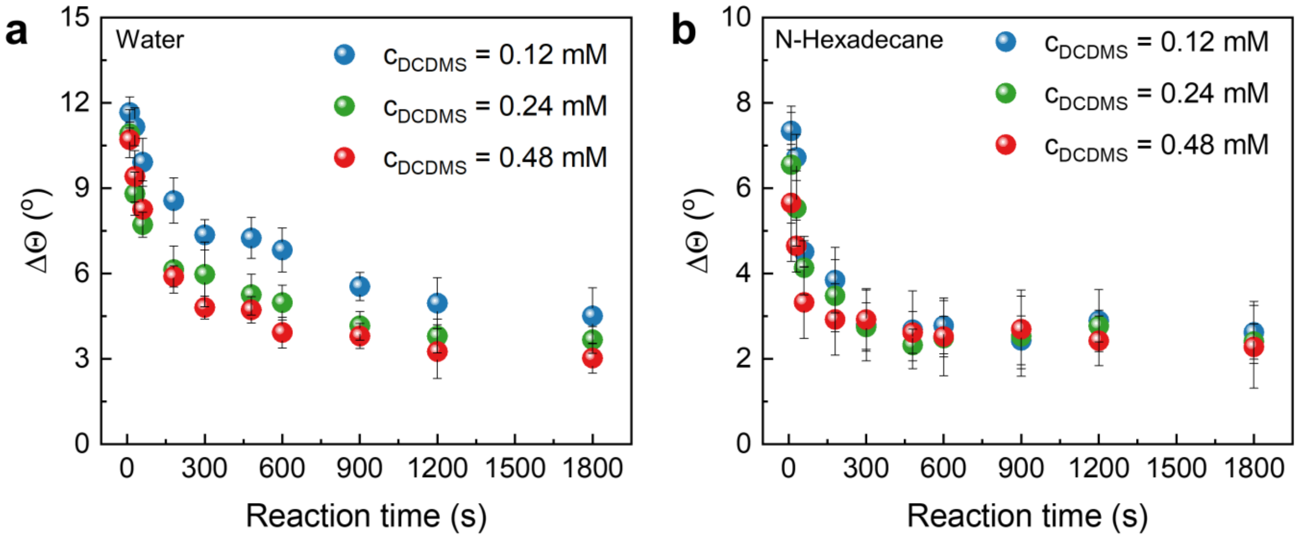

Figure S4 Influence of DCDMS concentration on grafting kinetics of PDMS brushes on silicon wafer. a) Contact angle hysteresis ($\Delta\Theta = \Theta_{ACA} - \Theta_{RCA}$) of water on PDMS brushes as function of grafting time and DCDMS concentration. b) Contact angle hysteresis ($\Delta\Theta$) of n-hexadecane on PDMS brushes as function of grafting time and DCDMS concentration. The water concentration (c_{water}) in toluene was 0.024 mM. The DCDMS concentration (c_{DCDMS}) was 0.12 mM (blue), 0.24 mM (green), or 0.48 mM (red). The grafting kinetics is accelerated with higher DCDMS concentration.

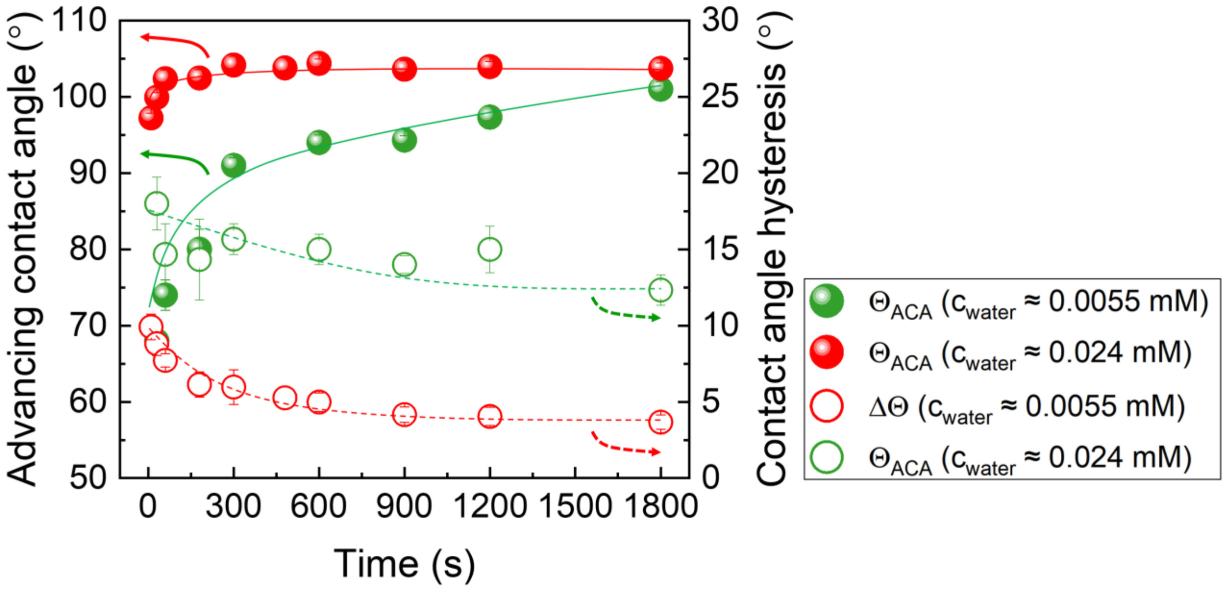

Figure S5 Influence of water concentration on the grafting process of PDMS brush. With a low water concentration (0.0055 mM) in toluene, the grafting speed is much slower than that when the water concentration is 0.024 mM. After reacting for 30 min, the PDMS brushes prepared from a low water concentration exhibit contact angle hysteresis of $\Delta\Theta \approx 13^\circ$ which is much higher than $\Delta\Theta \approx 4^\circ$ of the brushes grafted from $c_{water} \approx 0.024$ mM. Therefore, the higher concentration of water can effectively accelerate the reaction and reduce grafting time.

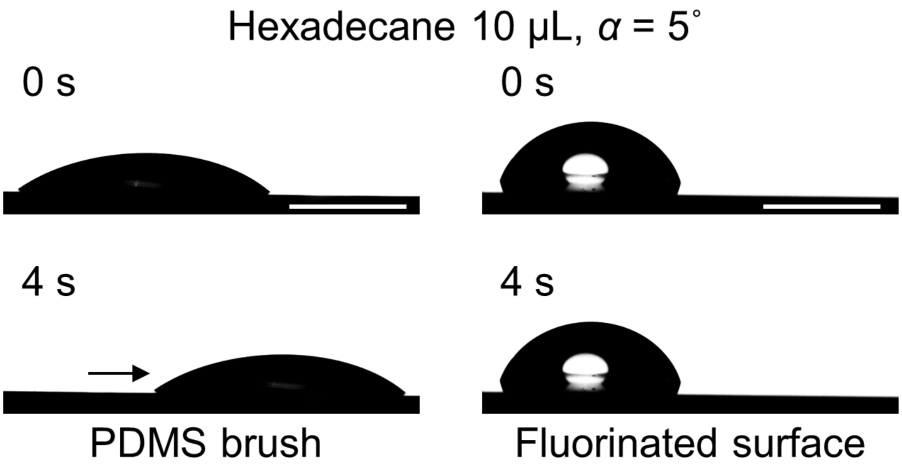

Figure S6 Sliding of n-hexadecane on hydrophobic coatings. 10 μL n-hexadecane droplet slides more easily on PDMS brushes than on the fluorinated surface. The fluorinated surface was modified with 1H,1H,2H,2H-perfluorooctyltrimethoxysilane. Tilt angle: 5°. Scale bar: 2 mm.

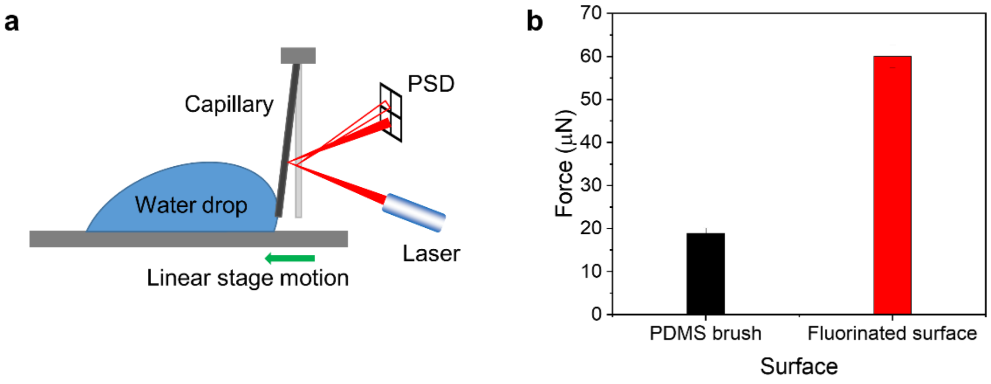

Figure S7 Measurement of lateral adhesion force of water droplet on surfaces. a) A drop of liquid is placed on a solid substrate mounted on a linear stage driven by a step motor.[2] A laser beam incident on the capillary is reflected to a position-sensitive detector (PSD). The contact width between the drop of liquid and the solid surface (orthogonal to the direction of motion) and contact length (parallel to the direction of motion) are simultaneously monitored by cameras (not shown). b) The lateral adhesion force of a water droplet (5 μL) on PDMS brushes and fluorinated surface. The relative moving velocity of the drop is 250 μm/s.

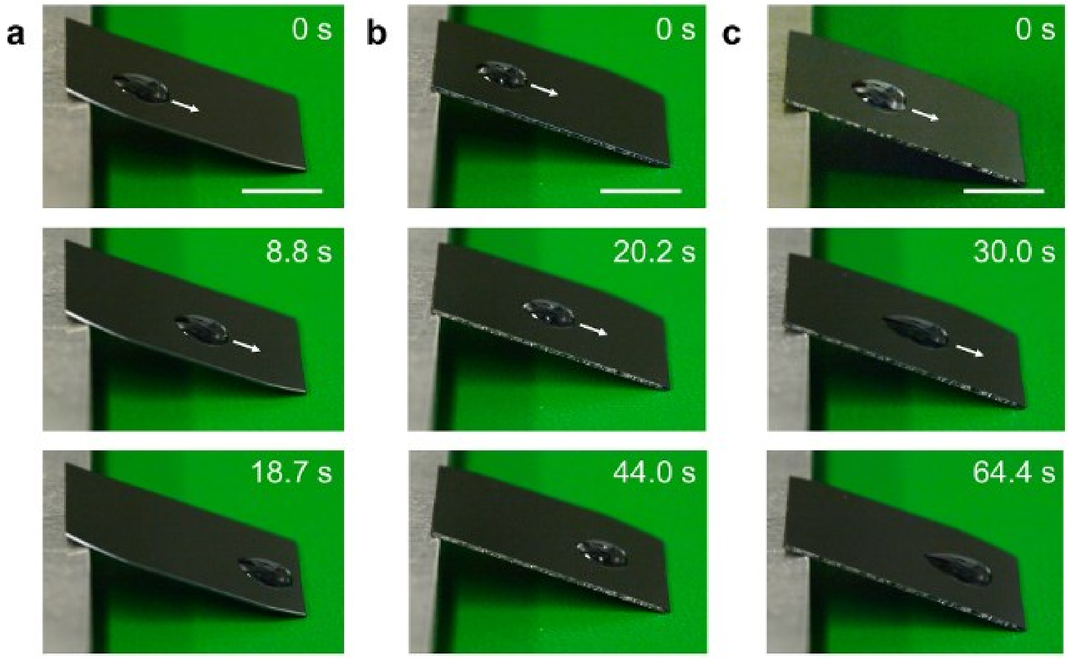

Figure S8 Sliding of liquid drops (30 μL) with high viscosity on the PDMS brush coated surface. The liquids are (a) poly(propylene glycol) (Mn ~725, viscosity: 115 cP), (b) poly(ethylene glycol)-block-poly(propylene glycol)-block-poly(ethylene glycol) (Mn ~2,000, viscosity: 325 cP), (c) poly(ethylene glycol)-block-poly(propylene glycol)-block-poly(ethylene glycol) (Mn ~4,400, viscosity: 1,200 cP). Tilt angle: 20°. Scale bar: 1 cm.

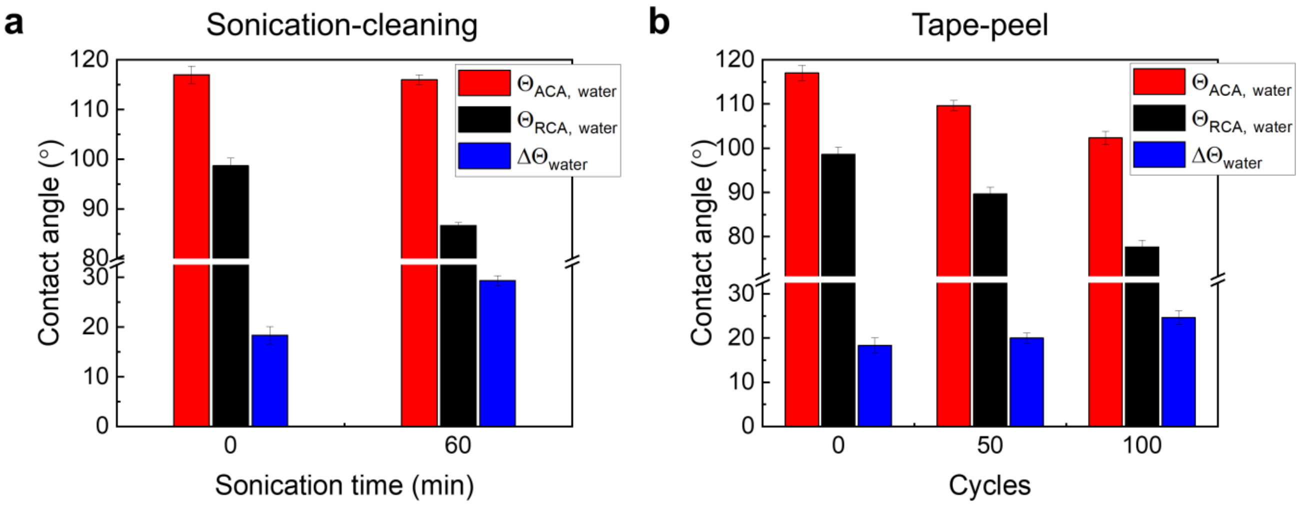

Figure S9 Mechanically robustness of fluorinated surface resisting sonication and tape-peel treatments. a) Advancing (Θ_{ACA}), receding (Θ_{RCA}) contact angle, and contact angle hysteresis ($\Delta\Theta$) of water on the fluorinated surface as function of sonication-cleaning time. The fluorinated surface was coated with 1H,1H,2H,2H-perfluorooctyltrimethoxysilane. b) Advancing (Θ_{ACA}), receding (Θ_{RCA}) contact angle, and contact angle hysteresis ($\Delta\Theta$) of water on the fluorinated surface as function of tape-peel test cycles. The receding contact angle of water decreases from 99° to 78° and the contact angle hysteresis increases from 18° to 25° after 100 peel-tests.

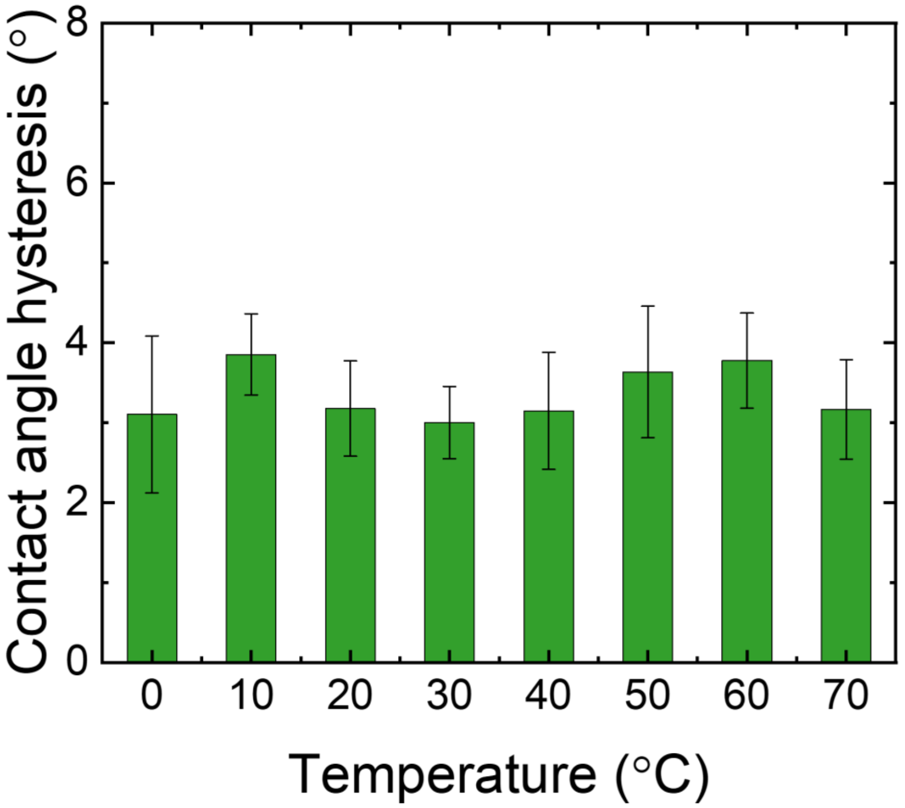

Figure S10 Stable wetting performance of PDMS brushes independent of temperature. Contact angle hysteresis of water on PDMS brushes was measured in a wide temperature window (from 0 °C to 70 °C). The test was done in a closed chamber with saturated water vapor, so that the evaporation or condensation effects of water have less influence on the wetting property measurements. The PDMS brushes exhibit a constant and low water adhesion independent of temperature.

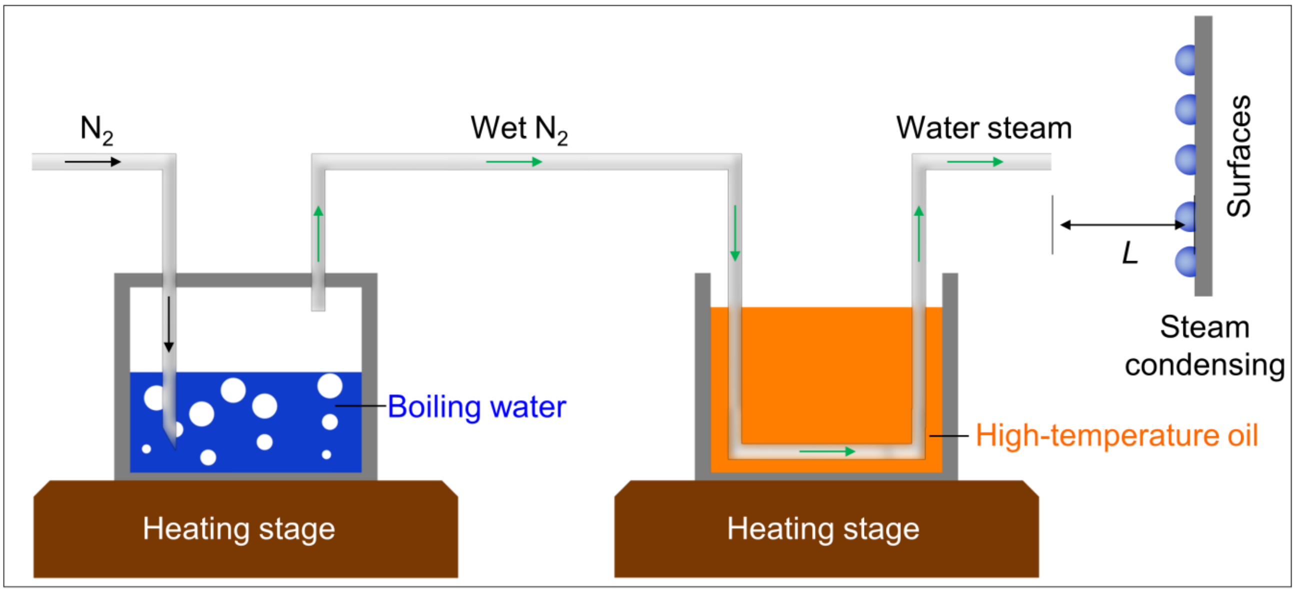

Figure S11 Schematic of the drop sliding erosion test. The setup was used to characterize the durability of PDMS brushes under high temperature (70 °C) and supersaturated water vapor treatment. Nitrogen was bubbled through boiling water with a flux of 0.8 ± 0.1 L min^{-1}. Then, the wet nitrogen was heated to 200 °C in a spiral circular copper tube. The PDMS brush coated surface was located in front of the outlet of the N_2 flux. Through changing the distance between surface and outlet, the vapor temperature can be controlled. The vapor temperature at the position of surface was determined by a mercury thermometer with a measuring range of 200 °C.

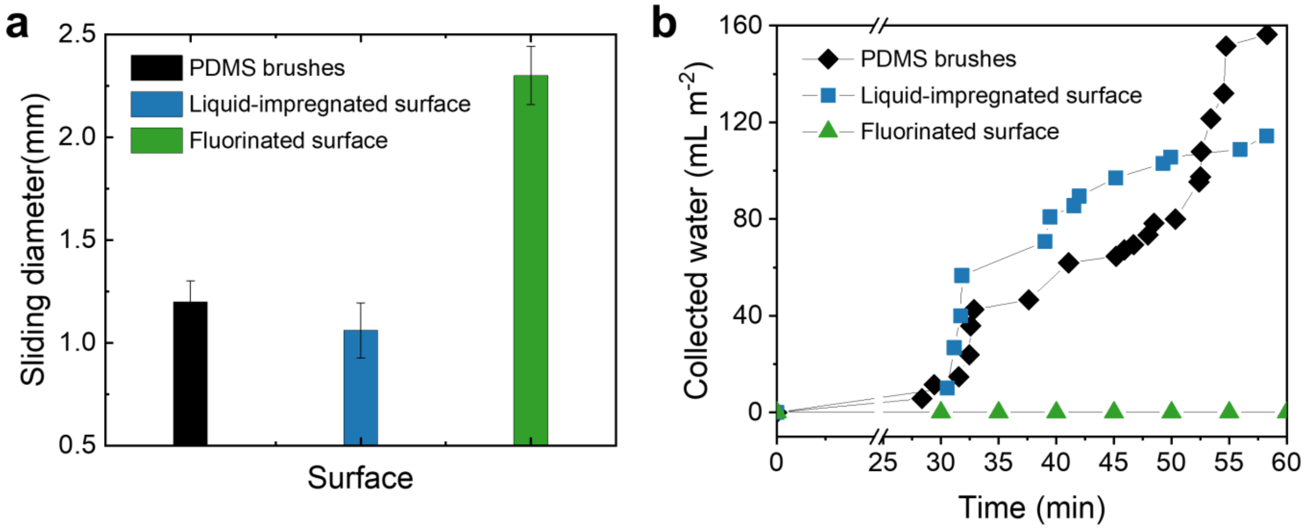

Figure S12 Mobility of condensed water droplets on PDMS brushes. a) The minimal sliding sizes of water droplets on different vertical surfaces (α = 90° fixed tilt angle) such as PDMS brushes, silicone oil impregnated PDMS brush surface (liquid-impregnated surface), and fluorinated surface. Condensed water droplets slid off the vertical PDMS brushes with a diameter (D) around 1.2 mm, a little bit smaller than that on liquid-impregnated surface (D = 1.0 mm). In contrast, the minimal sliding size of water drops on the fluorinated surface is much bigger (D = 2.3 mm). b) Water-collection efficiency (water volume slid off per square meter surface) on different vertical surfaces such as PDMS brushes, liquid-impregnated surface, and fluorinated surface at 0 °C (room temperature: 20 ± 1 °C, relative humidity: 80 ± 5%). PDMS brushes present the highest water-collection efficiency in one hour. Requiring big droplets for sliding, no water was collected by sliding at the bottom of the fluorinated surfaces when condensed for 1 hour.

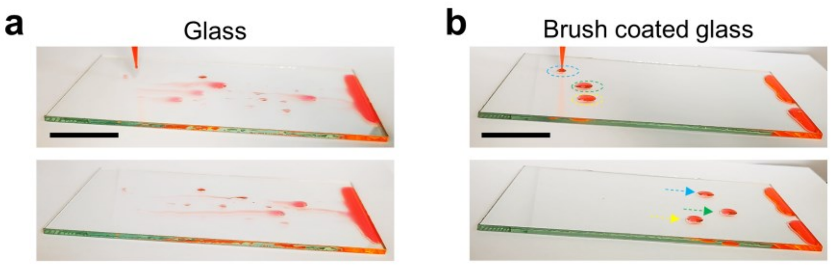

Figure S13 Large-scale fabrication of PDMS brushes on glass. a) Optical images show the pinning effect of toluene drops on window glass (20 × 10 cm^2) plate without PDMS brushes. b) Optical images show the sliding of toluene drops on window glass plate after coating PDMS brushes. Toluene drops are labeled with Nile red. Scale bar (a and b): 4 cm.

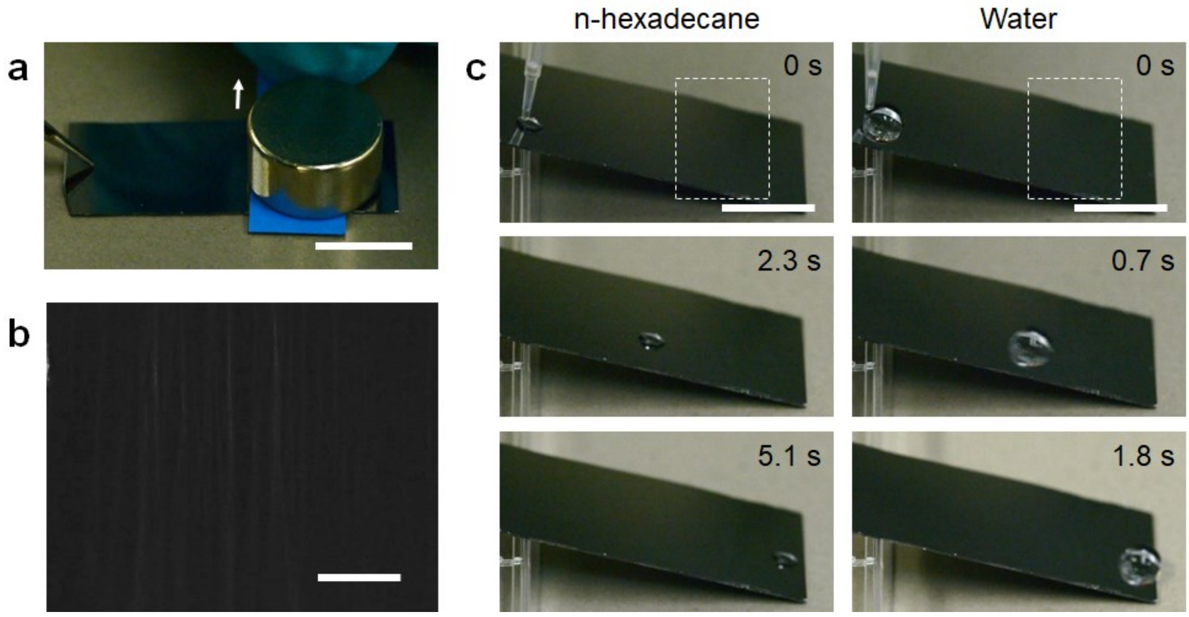

Figure S14 Abrasion test. a) A PDMS brush coated silicon wafer was rubbed with a sandpaper (1000 mesh) under the pressure of 1.1 kPa along the direction of the arrow. Scale bar: 1 cm. b) Digital image shows the scratches on the surface after abrasion for two times. Scale bar: 1 mm. c) Sliding of n-hexadecane (5 μL) and water (10 μL) droplets on the surface after sand paper (1000 mesh) abrasion. The dashed frame marks out the abrasion position. Scale bar: 1 cm.

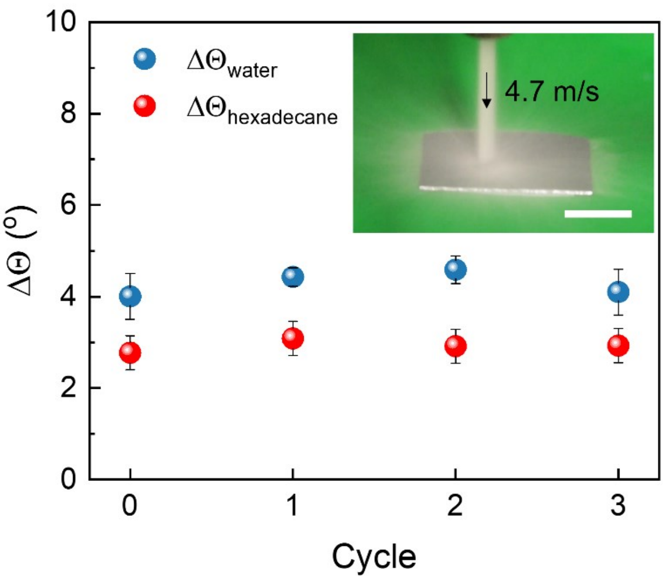

Figure S15 Stability of the surface resisting water jet impact. A water jet with mean velocity of 4.7 m/s was applied to impact the surface three times for 1 s. After the treatment, the wetting property of the surface remains unchanged with $\Delta\Theta_{water}$ = 4° ± 1° and $\Delta\Theta_{hexadecane}$ = 2.7° ± 1°. Inset shows the impacting of water jet on the surface. Scale bar: 1 cm.

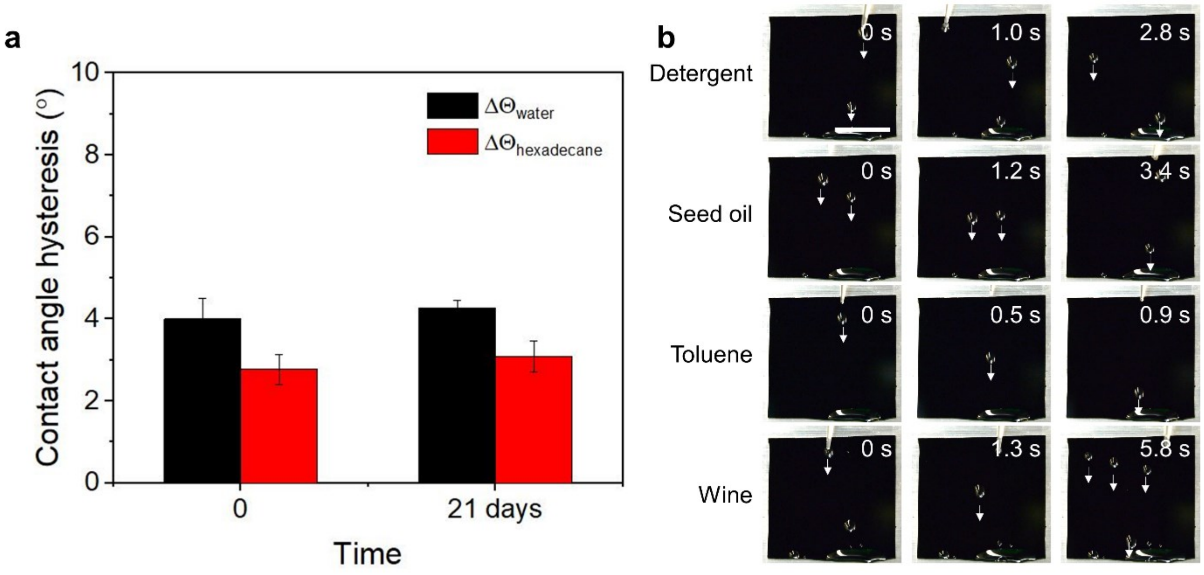

Figure S16 Real outdoor environment test. a) Contact angle hysteresis (ΔΘ) of water and n-hexadecane on PDMS brush coated silicon wafer after being placed in real outdoor environment for different time. b) Images show the sliding of various liquid drops on the PDMS brush coated surface after 21 days outdoor. Scale bar: 2 cm.

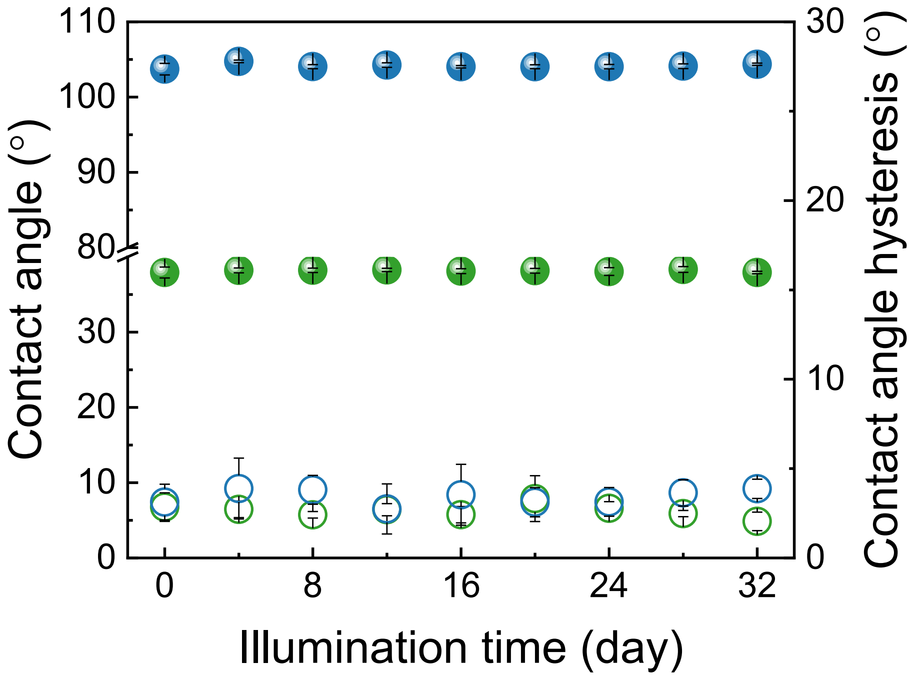

Figure S17 UV illumination resistance of PDMS brushes. Water (Θ_{ACA}: ● and $\Delta\Theta$: ○) and n-hexadecane (Θ_{ACA}: ● and $\Delta\Theta$: ○) repellency of the PDMS brushes versus UV illumination time. PDMS brushes present perfect wetting performance for at least 32 days under UV illumination. Wavelength: 365 nm. Intensity: 5 mW cm^{-2}.

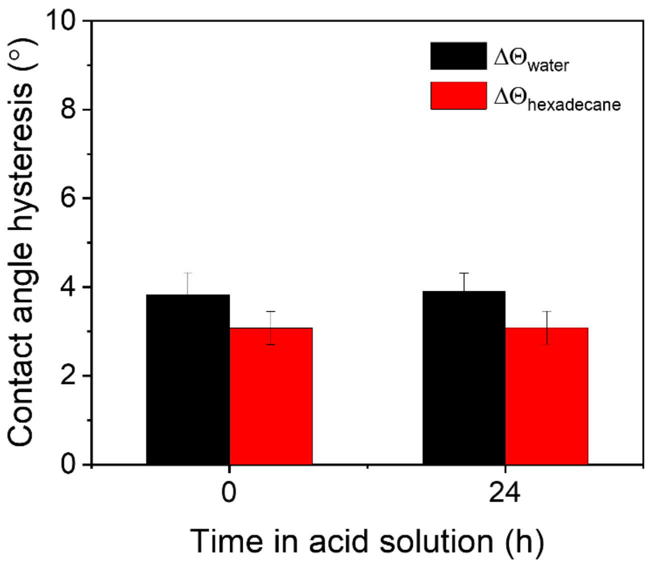

Figure S18 Stability of PDMS brushes in acid solution. HCl solution with pH = 1 was used. The surface is very stable even after immersing in acid solution for 24 hours. The wetting property remains the same with the original one. However, the surface is not stable and becomes hydrophilic after being immersed in the base solution. This might be caused by the chemical composition of the PDMS brushes, in which –Si–O– can react with the OH^-.

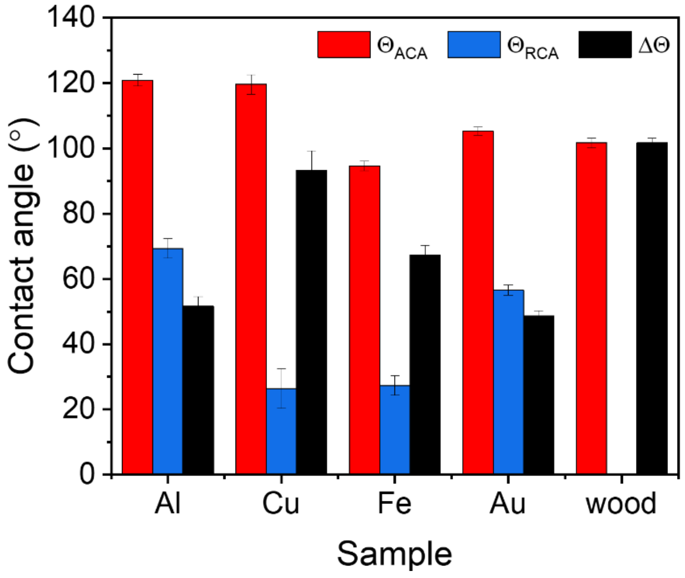

Figure S19 Advancing and receding contact angles (Θ_{ACA} and Θ_{RCA}) and contact angle hysteresis ($\Delta\Theta$) of water on various surface coated with PDMS brushes.

Movie S1 Fast and spontaneous grafting of PDMS brushes on glass surface. No residual stains were left. The induced self-repellency towards the reactant solution implies that no extra washing procedures are necessary to clean the surface, which reduces solvent waste.

Movies S2 Dropwise condensation of toluene on PDMS brushes. Condensed toluene can slide fast and easily on the PDMS brushes**.**

Movie S3 Washing of detergent stain on the PDMS brushes. The detergent can be completely cleaned by washing with less water flux. After cleaning water droplets slide easily on the surface.

Reference

[1] J. Ally, E. Vittorias, A. Amirfazli, M. Kappl, E. Bonaccurso, C. E. McNamee, H. J. Butt, *Langmuir* **2010**, *26*, 11797.

[2] N. Gao, F. Geyer, D. W. Pilat, S. Wooh, D. Vollmer, H.-J. Butt, R. Berger, *Nat. Phys.* **2017**, *14*, 191.
